# Hybrid immunity and protection against infection during the Omicron wave in Malta

**DOI:** 10.1080/22221751.2022.2156814

**Published:** 2023-01-02

**Authors:** John Paul Cauchi, Ausra Dziugyte, Maria-Louise Borg, Tanya Melillo, Graziella Zahra, Christopher Barbara, Jorgen Souness, Steve Agius, Neville Calleja, Charmaine Gauci, Pauline Vassallo, Joaquin Baruch

**Affiliations:** a Infectious Disease Prevention and Control Unit (IDCU), Health Promotion and Disease Prevention, Msida, Malta; b Molecular Diagnostics Pathology Department, Mater Dei Hospital, Msida, Malta; c Mater Dei Hospital, Msida, Malta; d Health Information and Research, Msida, Malta; e Ministry for Health, Superintendent of Public Health, Msida, Malta; f EPIET Programme, European Centre for Disease Prevention and Control, Solna, Sweden

**Keywords:** COVID-19, vaccines, hybrid immunity, public health, omicron (B.1.1.529)

## Abstract

By December 2021, administration of the third dose of COVID-19 vaccinations coincided with the spread of the Omicron variant in Europe. Questions had been raised on protection against infection conferred by previous vaccination and/or infection. Our study population included 252,433 participants from the COVID-19 vaccination registry in Malta. Data were then matched with the national testing database. We collected vaccination status, vaccine brand, vaccination date, infection history, and age. Using logistic regression, we examined different combinations of vaccine dose, prior infection status and time, and the odds of infection during the period when the Omicron variant was the dominant variant in Malta. Participants infected with Sars-Cov-2 prior to the Omicron wave had a significantly lower odds of being infected with the Omicron variant. Additionally, the more recent the infection and the more recent the vaccination, the lower the odds of infection. Receiving a third dose within 20 weeks of the start of the Omicron wave in Malta offered similar odds of infection as receiving a second dose within the same period. Time since vaccination was a strong determinant against infection, as was previous infection status and the number of doses taken. This finding reinforces the importance of future booster dose provision especially to vulnerable populations.

## Introduction

Vaccines are a critical tool to reduce the burden of coronavirus disease (COVID-19), caused by the severe acute respiratory syndrome coronavirus 2 (SARS-CoV-2). Public health institutions have also questioned the degree to which getting infected by SARS-CoV-2 would affect immunity, and for how long, especially in vaccinated people.

The first infection of COVID-19 in Malta was reported on 7th March 2020. Since then, Malta has gone through several waves of COVID-19, with the highest number of cases reported during the Omicron wave on December 2021 and January 2022. In addition, Malta had one of the fastest vaccination rollouts in the world [1], and within each wave vaccination coverage has increased. To date, COVID-19 vaccine coverage reached over 86% for two doses and over 67% for three doses [2]. Over 50% of those above 65 years of age have taken four doses [3].

Since November 2021, the Omicron SARS-Cov-2 variant spread rapidly around the world. In Malta, it was first detected in early December 2021, reaching over 80% prevalence in the week starting 20th December 2021 [4]. Compared to previous variants (Alpha and Delta) Omicron has been found to be more infectious, but less virulent, including when adjusted for vaccination and previous infection [5–7].

Andeweg et al. studied the effects of COVID-19 vaccination and previous SARS-CoV-2 infections, and found that they offer relatively low protection against Omicron infection [8], with a greater risk of infectivity and breakthrough [9]. Higher protection was observed against Omicron in individuals with both vaccination and previous infection, compared to either one, and protection since infection or vaccination decreased over time, also corraborated through a systematic review and meta-regression carried out by Feikin et al. [10]. This finding was been corroborated by Altarawneh et al., who also concluded that hybrid immunity from prior infection and recent booster vaccination confers strong protection against Omicron infection [11]. Vaccination, they conclude, enhances protection of those with a prior infection.

## Objective

This study, carried out on large cohort of people in Malta aims to understand vaccine effectiveness through a combination of vaccination, infection, and time. For ease of reference, the variants mentioned are referred to using WHO labels not their Pango lineage: Alpha (B.1.1.7), Beta (B.1.351), Delta (B.1.617.2) and Omicron (B.1.1.529) [12]. Similarly, we will be using the popular names for vaccines included in this study: Pfizer/Biontech (BNT162b2), Oxford-Oxford-AstraZeneca (ChADOx1 nCoV-19), Janssen (Ad26.COV2) and Moderna (mRNA-1273).

The main objective of our study was to evaluate the association between different combinations and timing of previous SAR-CoV-2 infections and vaccinations, with SAR-CoV-2 infection during the Omicron variant period in Malta, within the vaccinated Maltese population.

## Materials and methods

No ethical approval was necessary for this study as data collected were aggregate data from our surveillance systems, and results cannot be traced back to an individual. No funding was provided for this study.

### Study design and study subjects

The study is a retrospective cohort study, with study participants being all validated vaccinees in Malta’s COVID-19 vaccination registry between 27th December 2020 and 8th March 2022. The following criteria were used for inclusion of participants in our study (Supplementary Table 3).

### Inclusion criteria and data description

The following inclusion criteria applied in this study:

- Vaccinations between the 27th of December 2020 and 8th March 2022
- Vaccines received: Pfizer/Biontech, Oxford-AstraZeneca, Janssen, and/or Moderna
- Individuals alive on 8th March 2022
- Received at least 2 doses of vaccines (or 1 dose of Janssen)
- Did not receive a third dose between 15th December 2021 and 8th March 2022

The 15th December 2021 date was selected as being the midpoint of the week when Omicron became the dominant (>80% prevalence) Sars-Cov-2 variant in Malta. 80% was selected as the cut-off point following the example set by Andrews et al. [13]. This was further confirmed on a sensitivity analysis to be a suitable date (Supplementary Table 4), since there was little change in results for the dates of December 8th, 15th and December 22nd (making December 15th a suitable midpoint). Thorough quality checks were carried out on the vaccination registry database ensuring congruent data which included 403,755 participants. In this study, the outcome of interest was infection with SARS-Cov-2 between 15th December 2021 and 8th of March 2022, when it was assumed most new infections would be of the Omicron variant, which became the dominant variant (over 80%) of all infections that week.

Reverse Transcriptase Polymerase Chain Reaction (RT–PCR) and Rapid Diagnostic Test (RDT) test results for COVID-19 were obtained through the national database of RT–PCR and RDT tests. Since the RDT tests utilized in Malta were of high specificity and sensitivity (see Appendix 2), they were taken as confirmative of COVID-19 infection if positive. The data included all COVID-19 tests carried out in Malta until 8th March 2022 – following this date, the uptake of self-testing kits at home accelerated and self-reporting of COVID-19 dropped significantly, making any data after 8th March 2022 unreliable for our study. In individuals with repeated infection, an infection “event” was considered to be the first registered COVID-19 infection occurring at least 60 days after the previous infection event, as per literature findings [14]. The first registered event was either a positive RT–PCR test (75% of events) or a positive RDT test (25%).

Participants’ vaccination status was ascertained from the national immunization registry for COVID-19. Four vaccines were considered for this study, as they were main vaccinations provided to the population: Pfizer/Biontech, Moderna, Janssen, and Oxford-AstraZeneca. These were grouped into two groups: mRNA vaccines (Pfizer/Biontech and Moderna) and Adenoviral vaccines (Oxford-AstraZeneca and Janssen). Genomic data at a patient level was scarce, and therefore population level data were used. Two time points were determined: patients admitted before the Omicron variant reached 80% at a national level, namely, the Delta and other variants wave, vs patients admitted after 80% of the national sequences tested positive to Omicron, namely, the Omicron wave. All sequencing data were ascertained by whole genome sequencing.

During analysis, an attempt was made to include severity as an outcome. Unfortunately, data on severity (including symptomatic and hospitalization) was not validated and could not be ascertained to be of sufficient quality to include in a population-level study. Consequently, a decision was made to exclude this parameter from our study.

**Figure 1.**
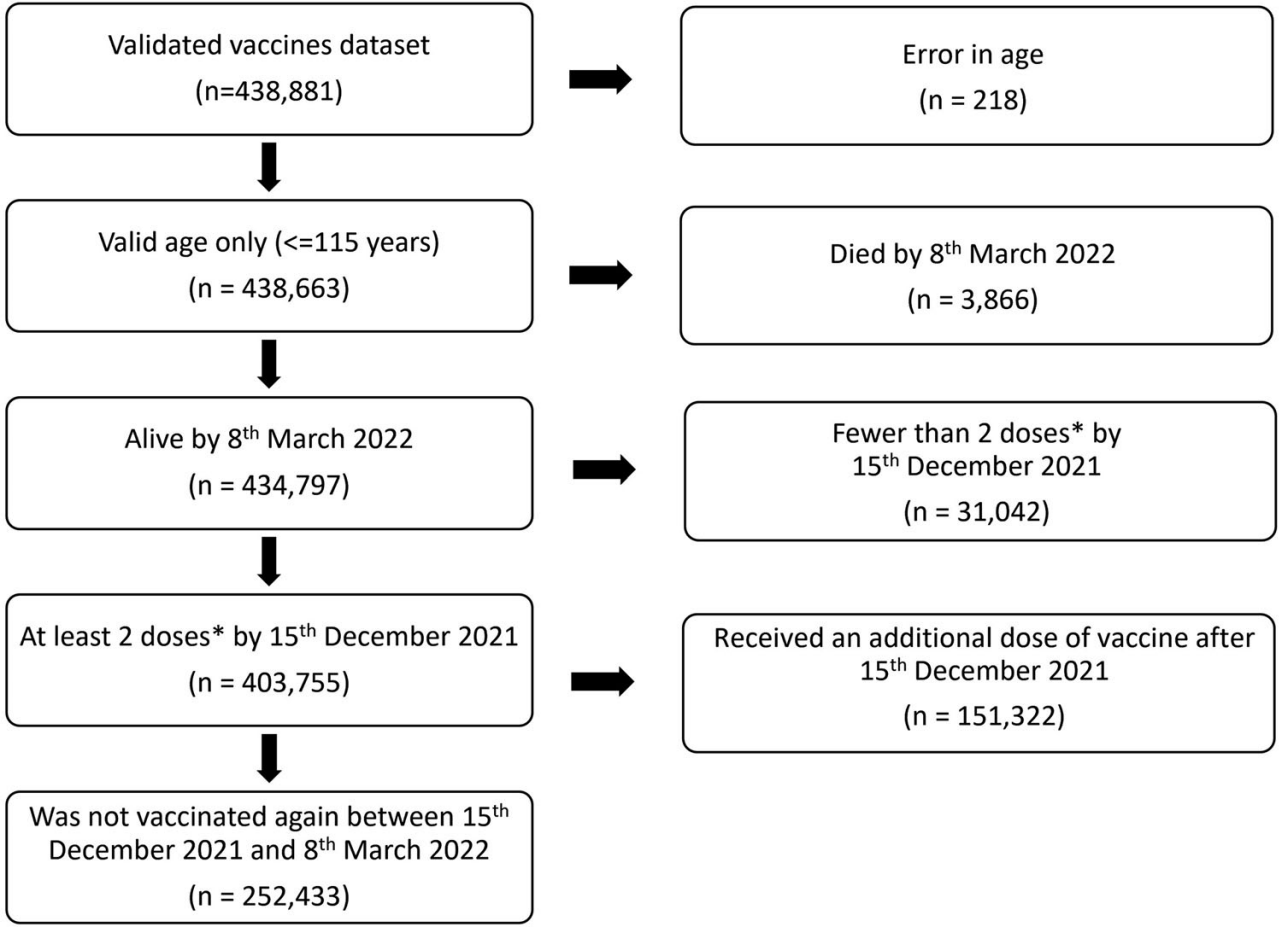
Flowchart showing inclusion criteria for cohort used in the study. *1 dose of Janssen vaccine is considered as a 2-dose regimen for this study (fully vaccinated).

The vaccination registry system registered 438,881 validated individuals from 27th December 2020 to 8th March 2022. Participants were excluded from the analysis as per the exclusion criteria, leaving a total of 403,755 possible participants (92.0%) in this study. After excluding those who got vaccinated between 15th December 2021 and 8th March 2022, 252,433 participants (57.5%) remained in the study, which is 48.9% of Malta’s residents [15] (Figure 1). By week 51, 2021, 82.4% of all variants sequenced were of the Omicron variant (Appendix 1), so the midpoint of the prior week (15th December 2021) was selected as a cut-off point.

### Statistical analysis

Data were cleaned and analysed using R software. Logistic regressions (R glm function) were fitted with infection during the Omicron wave as the outcome variable, and vaccination programme (number of doses), age (≤40 years, 41 years–70 years, 71 years+), infection pre-Omicron wave (Yes, No) and time (>20/<20 weeks since last vaccination/infection and 15th December 2021) as fixed effects. 20 weeks, or 5 months, were chosen as a cut-off for time as it was used in other studies measuring antibody responses to vaccination and infection [16–18]. Participants who had only taken two doses of vaccination prior to 15th December 2021, with the latest dose taken more than 20 weeks prior with no previous Sars-Cov-2 infection were considered the reference for these analyses.

## Results

### Demographics

Among 252,433 participants included in this study, the median age was 53 years (IQR 32 years–69 years). Out of the 252,433 study participants, 13,241 (5.2%) tested positive for COVID-19 during the Omicron wave (15th December 2021 to 8th March 2022) (Table 1). Of those who tested positive for COVID-19 during the Omicron wave, 537 (4.0%) had tested positive for COVID-19 before the Omicron wave. In our study, COVID-19 infection during the Omicron wave was most common in those aged 40 years and below (*n* = 7472, 56%), while those aged 71 years and above were the least likely to test positive for COVID-19 during the Omicron wave (*n *= 1388, 10.0%). The most common vaccination category until 8th March 2022 were participants who had taken 3 doses with the latest dose less than 20 weeks prior to 15th December 2021 and had no previous infection (*n* = 139,797, 55%). There were no participants registered as having taken 3 doses with the latest dose more than 20 weeks prior to 15th December 2021. Malta started administering the booster dose on 1st September 2021, less than 20 weeks before the Omicron wave.

**Table 1.** Descriptive table for the cohort included in the study, showing denominators and column percentages.

Characteristic					Outcome: Covid during Omicron	
Combination of doses, infection, and time				Overall *n* = 252,433	No *n *= 239,192 (94.8%)	Yes *n *= 13,241 (5.2%)
*Doses*	*Time from latest vaccination to 15th December 2021*	*Infection before 15th December 2021*	*Time from latest infection to 15th December 2021*			
2	>20 weeks	No	*NA*	61,690 / 252,433 (24%)	55,194 / 239,192 (23%)	6,496 / 13,241 (49%)
2	>20 weeks	Yes	>20 weeks	3,678 / 252,433 (1.5%)	3,399 / 239,192 (1.4%)	279 / 13,241 (2.1%)
2	>20 weeks	Yes	<20 weeks	802 / 252,433 (0.3%)	787 / 239,192 (0.3%)	15 / 13,241 (0.1%)
2	<20 weeks	No	*NA*	35,281 / 252,433 (14%)	33,737 / 239,192 (14%)	1,544 / 13,241 (12%)
2	<20 weeks	Yes	>20 weeks	1,554 / 252,433 (0.6%)	1,511 / 239,192 (0.6%)	43 / 13,241 (0.3%)
2	<20 weeks	Yes	<20 weeks	277 / 252,433 (0.1%)	275 / 239,192 (0.1%)	2 / 13,241 (<0.1%)
3	>20 weeks	No	*NA*	0 / 252,433 (0%)	0 / 239,192 (0%)	0 / 13,241 (0%)
3	>20 weeks	Yes	>20 weeks	0 / 252,433 (0%)	0 / 239,192 (0%)	0 / 13,241 (0%)
3	>20 weeks	Yes	<20 weeks	0 / 252,433 (0%)	0 / 239,192 (0%)	0 / 13,241 (0%)
3	<20 weeks	No	*NA*	139,797 / 252,433 (55%)	135,133 / 239,192 (56%)	4,664 / 13,241 (35%)
3	<20 weeks	Yes	>20 weeks	8,259 / 252,433 (3.3%)	8,069 / 239,192 (3.4%)	190 / 13,241 (1.4%)
3	<20 weeks	Yes	<20 weeks	1,095 / 252,433 (0.4%)	1,087 / 239,192 (0.5%)	8 / 13,241 (<0.1%)
Vaccination Categories						
*Doses*		*mRNA vaccines*	*Adenoviral vaccines*^†^			
2		2	0	59,088 / 252,432 (23%)	53,889 / 239,191 (23%)	5,199 / 13,241 (39%)
2		1	1	42,988 / 252,432 (17%)	39,863 / 239,191 (17%)	3,125 / 13,241 (24%)
2		0	2	1,206 / 252,432 (0.5%)	1,151 / 239,191 (0.5%)	55 / 13,241 (0.4%)
3		3	0	100,926 / 252,432 (40%)	97,779 / 239,191 (41%)	3,147 / 13,241 (24%)
3		2	1	47,770 / 252,432 (19%)	46,063 / 239,191 (19%)	1,707 / 13,241 (13%)
3		1	2 †	455 / 252,432 (0.2%)	447 / 239,191 (0.2%)	8 / 13,241 (<0.1%)
Age Categories						
40 years and below				93,768 / 252,433 (37%)	86,296 / 239,192 (36%)	7,472 / 13,241 (56%)
41–70 years				103,549 / 252,433 (41%)	99,168 / 239,192 (41%)	4,381 / 13,241 (33%)
71+ years				55,116 / 252,433 (22%)	53,728 / 239,192 (22%)	1,388 / 13,241 (10%)
Covid-19 infection prior to 15th December 2022^‡^						
No previous infection				236,768 / 252,433 (94%)	224,064 / 239,192 (94%)	12,704 / 13,241 (96%)
Alpha/Beta/Gamma infection (before 1st July 2021)				12,936 / 252,433 (5.1%)	12,441 / 239,192 (5.2%)	495 / 13,241 (3.7%)
Delta infection (after 1st July 2021)				2,729 / 252,433 (1.1%)	2,687 / 239,192 (1.1%)	42 / 13,241 (0.3%)

^†^ 2 adenoviral vaccines may refer to either two Oxford-AstraZeneca doses or one Janssen dose.

^‡^ In Malta the Delta variant became the dominant variant after 1st July 2021.

### Stratification by age group

When stratified by age groups (Table 2) a clear pattern emerges for all age groups, showing that both the number of vaccine doses received and previous infection are factors that contribute to an improvement on the protection against Omicron infection. Specifically, those with recent (<20 weeks) vaccination or recent (<20 weeks) infection or both have significant lower odds of getting infected during the Omicron period than those that had never been infected and were vaccinated with two doses more than 20 weeks before December 15th. For example, for those with 3 doses, with the third dose taken <20 weeks prior to December 15th and who had been infected <20 weeks prior to December 15th we obtained the following results across the three age groups: 40 years and below, OR = 0.14, CI = 0.04,0.33; 41 years–70 years, OR = 0.05, CI = 0.01,0.12; 71+ years, OR = 0.08, CI = 0.001,0.37 as compared to the reference category listed with OR = 1.0 (refer to Table 2 and compare across age groups for all other combinations). The 71+ years age group had the greatest protection against infection during Omicron (OR = 0.30; CI = 0.28, 0.32, compared to the reference group of 40 years and below, 1.00), with 70% lower odds of getting infected with Omicron (Supplementary Table 1).

**Table 2.** Univariate logistic regression, stratified by age groups.

Characteristic				Covid-19 Infection during Omicron								
				40 years and below			41 years–70 years			71+ years		
Combination of doses, infection, and time				OR	95% CI	*p*-value	OR	95% CI	*p*-value	OR	95% CI	*p*-value
*Doses*	*Time from latest vaccination to 15th December 2021*	*Infection before 15th December 2021*	*Time from latest infection to 15th December 2021*	*N = 93,768*	*N event = 7742*	*N = 103,549*	*N event = 4381*	*** ***	*N = 55,116*	*** ***	*N event = 1388*	*** ***
2	>20 weeks	No	*NA*	1.00 (Ref)	–	* *	1.00 (Ref)	–	* *	1.00 (Ref)	–	* *
2	>20 weeks	Yes	>20 weeks	0.71	0.61, 0.81	**<0.001**	0.66	0.50, 0.85	**0.002**	0.69	0.21, 1.68	0.47
2	>20 weeks	Yes	<20 weeks	0.16	0.08, 0.28	**<0.001**	0.17	0.06, 0.38	**<0.001**	0.00		0.95
2	<20 weeks	No	*NA*	0.39	0.37, 0.42	**<0.001**	0.29	0.25, 0.34	**<0.001**	0.96	0.60, 1.49	0.87
2	<20 weeks	Yes	>20 weeks	0.24	0.17, 0.33	**<0.001**	0.20	0.08, 0.41	**<0.001**	0.00	0.00, 0.07	0.95
2	<20 weeks	Yes	<20 weeks	0.08	0.01, 0.26	**<0.001**	0.00	0.00, 0.00	0.86	0.00		0.96
3	>20 weeks	No	*NA*	*NA*	*/*	*/*	*NA*	*/*	*/*	*NA*	*/*	*/*
3	>20 weeks	Yes	>20 weeks	*NA*	*/*	/	*NA*	*/*	/	*NA*	*/*	/
3	>20 weeks	Yes	<20 weeks	*NA*	*/*	/	*NA*	*/*	/	*NA*	*/*	/
3	<20 weeks	No	*NA*	0.61	0.57, 0.65	**<0.001**	0.28	0.26, 0.30	**<0.001**	0.61	0.50, 0.76	**<0.001**
3	<20 weeks	Yes	>20 weeks	0.31	0.24, 0.40	**<0.001**	0.16	0.13, 0.20	**<0.001**	0.54	0.37, 0.76	**<0.001**
3	<20 weeks	Yes	<20 weeks	0.14	0.04, 0.33	**<0.001**	0.05	0.01, 0.12	**<0.001**	0.08	0.00, 0.37	**0.013**
Vaccination Categories												
*Doses*		*mRNA vaccines*	*Adenoviral vaccines*^†^	*** ***	*** ***	*** ***	*** ***	*** ***	*** ***	*** ***	*** ***	*** ***
2		2	0	–	–		–	–		–	–	
2		0	2†	0.75	0.71, 0.79	**<0.001**	0.90	0.83, 0.99	**0.029**	1.12	0.39, 2.53	0.8
2		1	1	1.41	0.83, 2.26	0.2	0.38	0.27, 0.53	**<0.001**	0.80	0.13, 2.61	0.8
3		3	0	0.71	0.65, 0.77	**<0.001**	0.33	0.30, 0.36	**<0.001**	0.64	0.53, 0.78	**<0.001**
3		1	2†	0.71	0.65, 0.78	**<0.001**	0.31	0.29, 0.34	**<0.001**	0.52	0.35, 0.76	**<0.001**
3		2	1	0.00		>0.9	0.20	0.03, 0.63	**0.023**	0.46	0.18, 0.96	0.062
Covid-19 infection prior to 15th December 2022^‡^												
No previous infection				–	–		–	–		–	–	
Alpha/Beta/Gamma infection (before 1st July 2021)				0.69	0.61, 0.77	**<0.001**	0.60	0.50, 0.70	**<0.001**	0.85	0.63, 1.11	0.2
Delta infection (after 1st July 2021)				0.25	0.17, 0.35	**<0.001**	0.25	0.13, 0.42	**<0.001**	0.21	0.03, 0.64	**0.026**

^†^ 2 adenoviral vaccines may refer to either two Oxford-AstraZeneca doses or one Janssen dose.

^‡^ In Malta the Delta variant became the dominant variant after 1st July 2021.

### Stratification by vaccination groups

**Figure 2.**
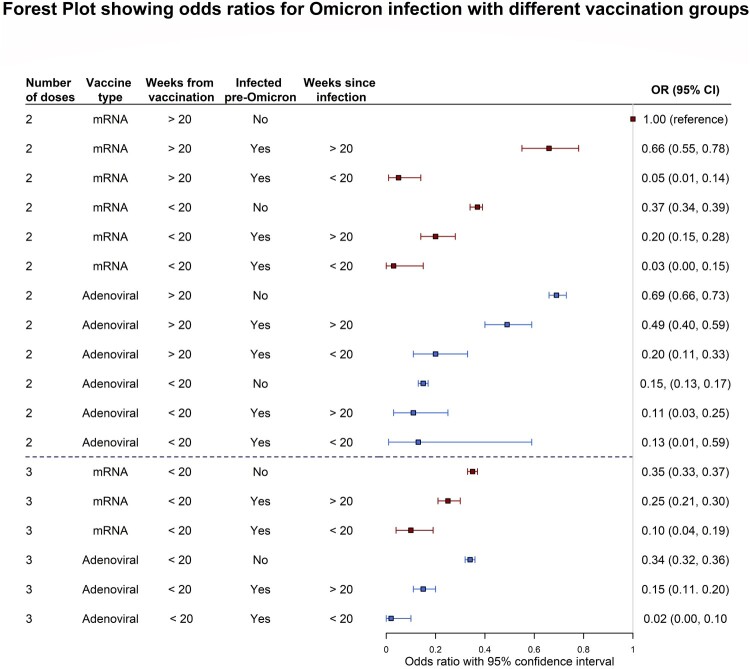
Forest plot showing the Odds Ratios for Omicron infection with different vaccination groups described in Table 3, adjusted for age group. Red and blue indicate mRNA vaccines and Adenoviral vaccines respectively. NB: The “3 Adenoviral” regimen indicates 2 Adenoviral doses equivalent and 1 mRNA booster. Figure Footnote: The reference category is indicated by the vertical line in the graph, showing a 1.00 OR. This refers to the 2 mRNA vaccines, >20 weeks, no infection category (weakest immunity conferred).

Multiple logistic regression was carried out for vaccination groups (Table 3 and Figure 2), adjusting for agegroup since vaccine regimens differed significantly between one age group and another. As in the rest of this paper, two adenoviral (AdX) vaccines can refer to either participants who received two Oxford-AstraZeneca vaccines, or one Janssen vaccine. The reference categories here were people who did not get infected prior to 15th December 2021 and who were vaccinated more than 20 weeks prior to 15th December 2021. Note that for the third dose, there were no participants receiving a third dose more than 20 weeks prior to 15th December 2021.

**Table 3.** Multiple Logistic Regression for vaccination groups adjusted for age groups.

Combination of doses, infection, and time				OR	95% CI	*p*-value
*Vaccination*	*Time from latest vaccination to 15th December 2021*	*Infection before 15th December 2021*	*Time from latest infection to 15th December 2021*	*N = 250,771*	* *	*N event = 13,178*
2 mRNA	>20 weeks	No	*NA*	1.00 (Ref)	–	* *
2 mRNA	>20 weeks	Yes	>20 weeks	0.66	0.55, 0.78	**<0.001**
2 mRNA	>20 weeks	Yes	<20 weeks	0.05	0.01, 0.14	**<0.001**
2 mRNA	<20 weeks	No	*NA*	0.37	0.34, 0.39	**<0.001**
2 mRNA	<20 weeks	Yes	>20 weeks	0.20	0.14, 0.28	**<0.001**
2 mRNA	<20 weeks	Yes	<20 weeks	0.03	0.00, 0.15	**<0.001**
2 Adx	>20 weeks	No	*NA*	0.69	0.66, 0.73	**<0.001**
2 Adx	>20 weeks	Yes	>20 weeks	0.49	0.40, 0.59	**<0.001**
2 Adx	>20 weeks	Yes	<20 weeks	0.20	0.11, 0.33	**<0.001**
2 Adx	<20 weeks	No	*NA*	0.15	0.13, 0.17	**<0.001**
2 Adx	<20 weeks	Yes	>20 weeks	0.11	0.03, 0.25	**<0.001**
2 Adx	<20 weeks	Yes	<20 weeks	0.13	0.01, 0.59	**0.042**
3 mRNA	>20 weeks	No	*NA*	*NA*	*/*	*/*
3 mRNA	>20 weeks	Yes	>20 weeks	*NA*	*/*	*/*
3 mRNA	>20 weeks	Yes	<20 weeks	*NA*	*/*	*/*
3 mRNA	<20 weeks	No	*NA*	0.35	0.33, 0.37	**<0.001**
3 mRNA	<20 weeks	Yes	>20 weeks	0.25	0.21, 0.30	**<0.001**
3 mRNA	<20 weeks	Yes	<20 weeks	0.10	0.04, 0.19	**<0.001**
2 Adx 1 mRNA	>20 weeks	No	*NA*	*NA*	*/*	*/*
2 Adx 1 mRNA	>20 weeks	Yes	>20 weeks	*NA*	*/*	*/*
2 Adx 1 mRNA	>20 weeks	Yes	<20 weeks	*NA*	*/*	*/*
2 Adx 1 mRNA	<20 weeks	No	*NA*	0.33	0.31, 0.36	**<0.001**
2 Adx 1 mRNA	<20 weeks	Yes	>20 weeks	0.15	0.11, 0.20	**<0.001**
2 Adx 1 mRNA	<20 weeks	Yes	<20 weeks	0.02	0.00, 0.10	**<0.001**
**Agegroup**						
40 years and below			1.00 (Ref)	–		
41–70 years				0.67	0.64, 0.70	**<0.001**
71+ years				0.44	0.41, 0.47	**<0.001**

^†^ Adx refers to either two Oxford-AstraZeneca doses or one Janssen vaccine dose. Here we have excluded mixed vaccine regiments (eg. 1 Adx and 1 mRNA) as they were too few in number and lacked power.

Compared to the reference, those who received a third dose before 15th December 2021 and had no previous infection had 65% (OR = 0.35, CI 0.33, 0.37) lower odds of getting an infection with Omicron up to the 8th of March 2022. Those who received the equivalent of two adenoviral doses however, even if more than 20 weeks prior to 15th December 2021, had 31% (OR = 0.69, CI 0.66:0.73) lower odds of becoming infected with Omicron.

Combined with infection, the protective effect is greatly amplified across all regimens (Figure 2). For example, those who took two mRNA vaccines and were infected with Omicron more than 20 weeks prior to 15th December 2021 had 34% (OR = 0.66, CI 0.55, 0.78) lower odds while those who took two adenoviral vaccines’ equivalent and were likewise infected more than 20 weeks prior had 51% (OR = 0.49, CI 0.40, 0.59) lower odds. This effect is further amplified with a recent infection with 20 weeks of 15th December 2021 for more recent infections, with 95% (OR = 0.05, CI 0.01, 0.14) and 80% (OR = 0.20, CI 0.11, 0.33) for two mRNA and two adenoviral vaccines’ equivalent respectively. Taking a third dose within 20 weeks of 15th December 2021 further increased this protective effect.

## Discussion

This study highlights the role of time, vaccination and infection against infection with the Omicron variant. The outcomes of this study inform public health institutions and policymakers on current and future vaccination strategies for COVID-19. These insights should prove increasingly important as the world enters a scenario where COVID-19 is increasingly being treated as an endemic disease [19,20], when additional booster doses being offered to the more vulnerable members of society.

### A matter of time

The outcomes of this study demonstrate that being vaccinated with three doses of vaccine offers greater protection against Omicron infection overall than two doses of vaccine. Receiving a third dose within 20 weeks of the start of the Omicron wave in Malta with no previous infection offered similar odds of infection as receiving a second dose within the same period, with no previous infection (Table 3). This applies for both mRNA and adenoviral regimens. This indicates that for those not previously infected, it is more a matter of timing since the last vaccination, rather than a matter of the number of doses, that has the stronger protective effect against Omicron. Being previously infected with COVID-19 also confers additional protection against infection during the Omicron period. However, the most impactful factor in conferring protection against infection is the timing of the most recent vaccination or infection (more or less than 20 weeks until 15th December 2021). For both infection and vaccination, less than 20 weeks until the start of the Omicron period conferred better protection. This finding matches the literature, which refers to waning immunity over time [21–24]. Hybrid immunity (infection and vaccination) also provides the higher protection than vaccination on its own, again matching literature on the subject [25].

### Age matters

Academic literature shows strong evidence of increasing age being a significant risk factor for infection increased severity of COVID-19 infection [26,27]. However, in this analysis belonging to an older age group compared to a younger age group lowered the odds of being infected during the Omicron period (Table 3). This seemingly contradictory result is most likely due to the greater vaccine booster uptake by older (and more vulnerable) people in Malta, priming their immunity during the Omicron wave [2]. Younger people were less likely to take up a third dose; by 15th December 2021, only 20.5% of all those 40 years and below (*n* = 19,251/93,769), had been vaccinated with a third dose. This can be contrasted to the 94.1% (*n* = 51,095/55,116) of all those aged 71+ years who had taken a third dose by that date (Supplementary Table 2). This might explain why the youngest age group had the higher odds of being infected by Omicron.

Additionally, when looking within age groups one finds that for those aged 41–70 years the odds ratios are lower than for the two other groups. This difference in odds ratios within one group and another could fit with the narrative of younger people exhibiting lower cautious behaviour compared to older people, while older people having a lower immunity overall, as the lowering of odds changes significantly within each group.

For those aged 71+ years, the results in the “2 doses” section are not statistically significant. The wide confidence ratios for the “2 doses” section points to low power (and numbers), which contrasts with the much tighter confidence intervals in the “3 doses” section. This points to Malta’s very successful uptake of the third booster dose by the 15th of December 2021. Additionally, this finding suggests that unless given a booster dose, people aged 71+ years do not have associated lower odds of infection, even when compared to those that had COVID-19 before. This finding is an important one and underlines the importance of providing additional boosters over time to the elderly and vulnerable segments of the population as an effective public health measure.

### Vaccination groups

**Figure 3.**
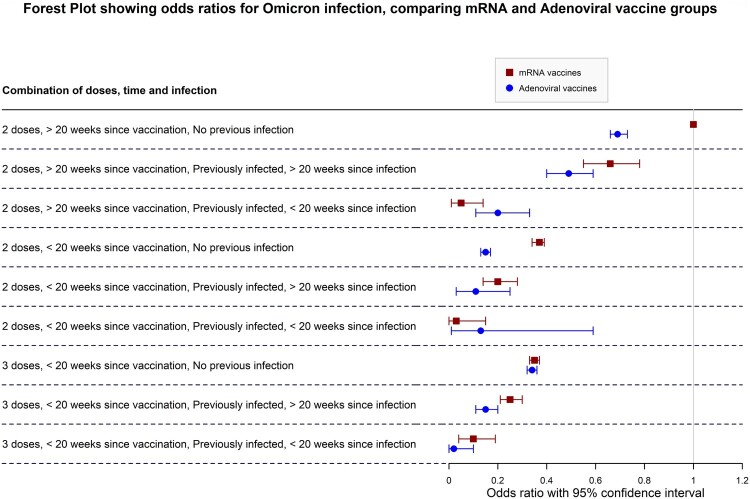
Forest plot comparing adenoviral to mRNA vaccine groups., adjusted for age group. NB: The “3 Adenoviral” regimen indicates 2 Adenoviral doses equivalent and 1 mRNA booster. Figure Footnote: The reference category is indicated by the vertical line in the graph, showing a 1.00 OR. This refers to the 2 mRNA vaccines, >20 weeks, no infection category (weakest immunity conferred).

Different vaccination regimens also seem to confer different protective effects against infection during the Omicron period, even after adjusting for age groups. While the magnitude of protection down different combination categories follows a similar pattern throughout, for two doses, adenoviral vaccines seem to offer greater protection against infection overall when compared to mRNA vaccine regimens if the last dose taken was more than 20 weeks prior to 15th December 2021. This may point to the different mode of action of adenoviral vaccines compared to mRNA vaccines, the former possibly offering better lasting protection overall compared to mRNA vaccine. This finding might also point to a longer-lasting protective effect of adenoviral vaccines in general compared to mRNA vaccines. This distinction disappears however with the third booster dose, with odds ratios of 65% lower odds for those with three mRNA vaccines and 67% (OR = 0.33, CI 0.31, 0.36) for those with two adenoviral and one mRNA vaccine. There is also considerable overlap of odds ratios between the two categories (Figure 3), indicating that this difference may not be significant except for those who took 2 doses and did not have a previous infection. For the three-dose category there is effectively no significant difference between both vaccine regimens, indicating that both are highly protective against Omicron, with recent infection improving the odds against infection. However, the confidence intervals for adenoviral vaccines in the 3rd dose categories suggest that perhaps mixing of vaccinations may provide a better protective effect against Omicron infection. This matches available literature on heterologous vaccine regiment effectiveness compared to homologous vaccination [28–30]. Unfortunately, those receiving other mixed vaccine regimens (for example, 1 mRNA + 2 adenoviral vaccines) were too few, limiting power so that we were not able to achieve any significant interpretations and findings, and have been excluded from Table 3 and the forest plots.

### Hybrid immunity

The strongest finding in this study is the protective effect of a combination of vaccination and previous infection against Omicron infection. The results of this study strongly affirm these findings, with lower odds ratios for those who had been previously infected with SARS-CoV-2 and had been vaccinated compared to those who received vaccination alone, for both two- and three-dose regimen. This is a subject of intense discussion in the literature, with evidence worldwide strongly supporting the greater immune response from vaccination and infection, as opposed to either one alone [11,31–33]. As aforementioned, the more recent the infection and/or vaccination, the greater the protective effect conferred against infection with the Omicron variant.

### Strengths and limitations

This study has several limitations. First, it is a cohort taken from Malta’s vaccination registry system, meaning that findings here are not related to unvaccinated individuals. As people have been getting with infected with COVID-19 and over 87% of the population are vaccinated, it becomes irrelevant to compare to an unvaccinated, uninfected group. Consequently, odds ratios here are not representative of vaccine efficacy, but are rather compared to the so-called “weakest immunity” scenario: people who received the equivalent of two doses of vaccine more than 20 weeks prior to the start of the Omicron period in Malta, with no previous infection. Here, therefore, we observe the protective effect against infection of one sub-category over another during the Omicron period.

Second, this dataset did not include sex, which could be an important confounder for immunity: a 2021 metanalysis by Bignucolo et al. found vaccine efficacy to be higher in men than in women [34]. Third, this study does not examine subvariants of Omicron prevalent in Malta up until 8th March 2022. However, it is safe to assume that the two variants present at the time were the BA.1 and BA.2 subtypes. Fourth, 25% of participants had a positive RDT test as a first indicator of an infection event and were included in this study as infected participants even if they were not confirmed as RT–PCR positive (although in most cases they were confirmed). However, RDT tests used in Malta were of very high specificity and sensitivity (Appendix 2). In addition, if these RDTs present a lower sensitivity than shown in Appendix 2, it would be safe to assume that this lower sensitivity would not be associated with the exposure – leading to non-differential misclassification. If the misclassification is non-differential, we would observe an increase in the width of the confidence interval, which would be corrected by our large sample size. Fifth, our genomic information came from population estimates, not from individual level data. This approach has been used before.

Sixth, in the Andrews et al., study the authors also included a cut-point of 80% for estimating the switch on a dominant variant, and we opted to follow their example [7]. This however left a limitation in the study where some people might have been infected with the Omicron infection prior to the 15th of December. In order to confirm the 15th of December as the ideal date, we carried out sensitivity analysis on the following dates as the cut-off point: 8th December 2021, 15th December 2021 and 22nd December 2021 (Supplementary Table 4). We found little difference in results for the three dates. We therefore opted for the 15th December which also coincides with the midpoint of the week when Omicron became the dominant variant in Malta [4]. Seven, while our study compares the odds of infection between different “immunity” groups, it is not a reflection of antibody levels. Therefore, we caution the reader not to interpret these results as a level of antibody response. Lastly, our study lacks a measure of severity of infection, which would have been interesting given the combination of time, vaccination and previous infection as a measure of this study.

Our study also has several strengths. First, our cohort is a large, mostly homogenous population of over 255,433 participants. This makes the findings robust. We also removed erroneous entries to ensure that the data were as clean and accurate as possible. Second, in Malta until the 8th of March 2022 self-testing kits were not easily available, and all private and public RDT and RT–PCR tests were reported to a centralized database, making the outcomes measured here comprehensive for the Maltese population. Third, Malta has a very high vaccine uptake compared to other countries, which makes such a study comparing the effects of two to three dose vaccinations and infection particularly robust.

## Conclusion

The outcomes of this study stress the importance of time in triggering an immune response and protective effect against infection by the Omicron variant. In this light, booster doses remain highly important regardless of infection status, especially when hybrid immunity is shown to be highly effective against subsequent infections by SARS-CoV-2. As most countries prepare to treat COVID-19 as an endemic rather than an epidemic disease, the findings of this study point to the importance of maintaining booster dose uptake by the more vulnerable members of society, especially considering new variants that may develop over time. Given the evidence on waning immunity over time, research on finding the critical best time to administer boosters becomes increasingly important [24,35]. There also remains a significant risk of getting infected with the Omicron (and possibly future) variants for vulnerable segments of the population in spite of vaccination status and previous infection; in this case, avoidance of exposure remains necessary especially during period of high infection incidence of COVID-19.

## Supplementary Material

Supplemental Material

## Appendices

### Appendix 1. Genetic sequencing of SARS-CoV-2 variants over time in Malta

The red line depicts the Delta sequences whereas the blue line depicts the Omicron sequences.

### Appendix 2. Rapid diagnostic tests

The following is a list of all Rapid Diagnostic Tests used in Malta. Note that percentages listed are specifications provided by the manufacturer.

**Table:**

Test	Sensitivity	Specificity
SARS-CoV-2 Rapid Antigen Test Biosencor Roche	95.50%	99.20%
MEDsan SARS-CoV-2 Antigen Rapid Test; MEDsan GmbH	92.50%	99.80%
AMP Rapid Test SARS-CoV-2 Ag; AMEDA Labordiagnostik GmbH	97.30%	100.00%
Clungene Rapid Test COVID-19 Antigen Rapid Test Cassette; Hangzhou Clongene Biotech Co., Ltd.	91.40%	100%
Coronavirus Ag Rapid Test Cassette (Swab); Healgen Scientific Limited Liability Company	98.32%	99.60%
COVID-19 (SARS-CoV-2) Antigen Test Kit (Colloidal Gold); Anhui Deep Blue Medical Technology Co., Ltd	95%	99%
NADAL COVID-19 Ag Test; Nal Von Minden GmbH	97%	98%
NOVA Test SARS-CoV-2 Antigen Rapid Test Kit (Colloidal Gold Immunochromatography); Atlas Link Technology Co. Ltd.	98.50%	99.40%
SARS-CoV-2 Ag Rapid Test; BioMaxima S.A.	95.70%	99.10%
SARS-CoV-2 Antigen Rapid Test Kit; Beijing Lepu Medical Technology Co., Ltd	92%	99.30%
Wondfo 2019-nCoV Antigen Test (Lateral Flow Method); Guangzhou Wondfo Biotech Co., Ltd.	96.20%	99.70%
Coronavirus Ag Rapid Test Cassette; Zhejiang Orient Gene Biotech	98.32%	99.60%
Influenza & COVID-19 Ag Combo Rapid Test Cassette (Swab); Zhejiang Orient Gene Biotech Co., Ltd	98.32%	99.60%
Panbio COVID-19 Ag Rapid Test Device (Nasal); Abbott Rapid Diagnostics Jena GmbH	91.40%	99.80%
Average values	95.52%	99.44%
