# Supplementary material for: Hybrid immunity and protection against infection during the Omicron wave in Malta
Source: Emerg Microbes Infect. 2023 Jan 2;12(1):e2156814. doi: 10.1080/22221751.2022.2156814 (PMC9817114; doi:10.1080/22221751.2022.2156814)
Supplement: Supplemental Material [file TEMI_A_2156814_SM5900.zip › Appendices.docx]

# Appendix 1: Genetic sequencing of SARS-CoV-2 variants over time in Malta


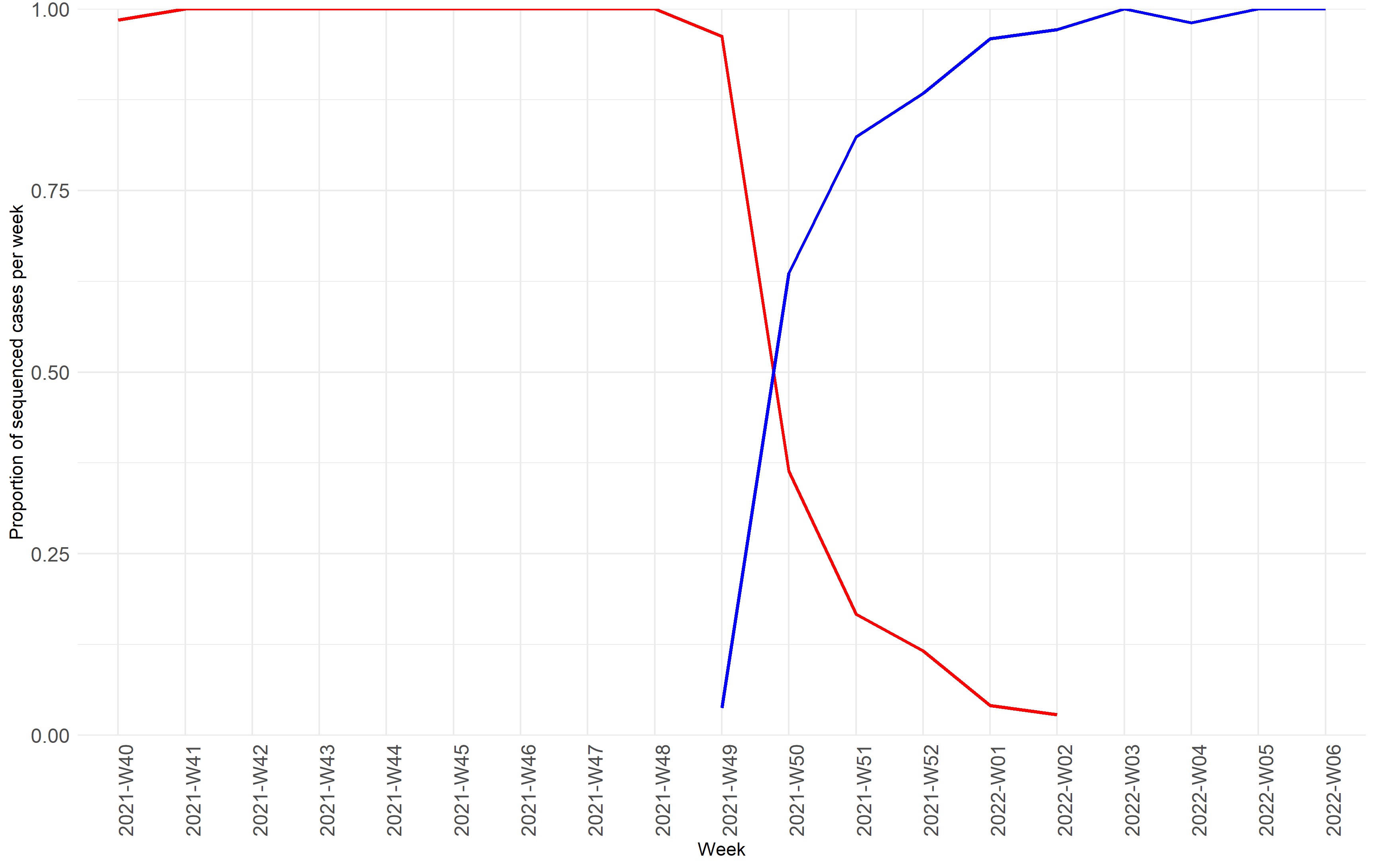


**Appendix 2: Rapid Diagnostic Tests**

The following is a list of all Rapid Diagnostic Tests used in Malta.

| Test | Sensitivity | Specificity |
| --- | --- | --- |
| SARS-CoV-2 Rapid Antigen Test Biosencor Roche | 95.50% | 99.20% |
| MEDsan SARS-CoV-2 Antigen Rapid Test; MEDsan GmbH | 92.50% | 99.80% |
| AMP Rapid Test SARS-CoV-2 Ag; AMEDA Labordiagnostik GmbH | 97.30% | 100.00% |
| Clungene Rapid Test COVID-19 Antigen Rapid Test Cassette; Hangzhou Clongene Biotech Co., Ltd. | 91.40% | 100% |
| Coronavirus Ag Rapid Test Cassette (Swab); Healgen Scientific Limited Liability Company | 98.32% | 99.60% |
| COVID-19 (SARS-CoV-2) Antigen Test Kit (Colloidal Gold); Anhui Deep Blue Medical Technology Co., Ltd | 95% | 99% |
| NADAL COVID-19 Ag Test; Nal Von Minden GmbH | 97% | 98% |
| NOVA Test SARS-CoV-2 Antigen Rapid Test Kit (Colloidal Gold Immunochromatography); Atlas Link Technology Co. Ltd. | 98.50% | 99.40% |
| SARS-CoV-2 Ag Rapid Test; BioMaxima S.A. | 95.70% | 99.10% |
| SARS-CoV-2 Antigen Rapid Test Kit; Beijing Lepu Medical Technology Co., Ltd | 92% | 99.30% |
| Wondfo 2019-nCoV Antigen Test (Lateral Flow Method); Guangzhou Wondfo Biotech Co., Ltd. | 96.20% | 99.70% |
| Coronavirus Ag Rapid Test Cassette; Zhejiang Orient Gene Biotech | 98.32% | 99.60% |
| Influenza & COVID-19 Ag Combo Rapid Test Cassette (Swab); Zhejiang Orient Gene Biotech Co., Ltd | 98.32% | 99.60% |
| Panbio COVID-19 Ag Rapid Test Device (Nasal); Abbott Rapid Diagnostics Jena GmbH | 91.40% | 99.80% |
| Average values | 95.52% | 99.44% |
