# Supplementary material for: Hybrid immunity and protection against infection during the Omicron wave in Malta
Source: Emerg Microbes Infect. 2023 Jan 2;12(1):e2156814. doi: 10.1080/22221751.2022.2156814 (PMC9817114; doi:10.1080/22221751.2022.2156814)
Supplement: Supplemental Material [file TEMI_A_2156814_SM5900.zip › Supplementary Table 1.docx]

**Supplementary Table 1 : Univariate Logistic Regression**

| **Characteristic** | | | | **COVID-19 Infection during Omicron** | | | |
| --- | --- | --- | --- | --- | --- | --- | --- |
| Combination of doses, infection, and time | | | | N | Odds Ratio | 95% CI | P value |
| ***Doses*** | ***Time from latest vaccination to 15th December 2021*** | ***Infection before 15^th^ December 2021*** | ***Time from latest infection to 15th December 2021*** | 252,433 |  |  |  |
| 2 | >20 weeks | No | *NA* |  | 1.00 (Ref) | — |  |
| 2 | >20 weeks | Yes | >20 weeks |  | 0.70 | 0.61, 0.79 | **<0.001** |
| 2 | >20 weeks | Yes | <20 weeks |  | 0.16 | 0.09, 0.26 | **<0.001** |
| 2 | <20 weeks | No | *NA* |  | 0.39 | 0.37, 0.41 | **<0.001** |
| 2 | <20 weeks | Yes | >20 weeks |  | 0.24 | 0.18, 0.32 | **<0.001** |
| 2 | <20 weeks | Yes | <20 weeks |  | 0.06 | 0.01, 0.19 | **<0.001** |
| 3 | >20 weeks | No | *NA* |  | *NA* | */* | */* |
| 3 | >20 weeks | Yes | >20 weeks |  | *NA* | */* | / |
| 3 | >20 weeks | Yes | <20 weeks |  | *NA* | */* | / |
| 3 | <20 weeks | No | *NA* |  | 0.29 | 0.28, 0.30 | **<0.001** |
| 3 | <20 weeks | Yes | >20 weeks |  | 0.20 | 0.17, 0.23 | **<0.001** |
| 3 | <20 weeks | Yes | <20 weeks |  | 0.06 | 0.03, 0.12 | **<0.001** |
| Vaccination Categories | | | | 252,433 |  |  |  |
| ***Doses*** | | ***mRNA vaccines*** | ***Adenoviral* vaccines*** |  |  |  |  |
| 2 | | 2 | 0 |  | 1.00 (Ref) | — |  |
| 2 | | 0 | 2* |  | 0.81 | 0.78, 0.85 | **<0.001** |
| 2 | | 1 | 1 |  | 0.50 | 0.37, 0.64 | **<0.001** |
| 3 | | 3 | 0 |  | 0.33 | 0.32, 0.35 | **<0.001** |
| 3 | | 1 | 2* |  | 0.38 | 0.36, 0.41 | **<0.001** |
| 3 | | 2 | 1 |  | 0.19 | 0.08, 0.35 | **<0.001** |
| Age Categories | | | | 252,433 |  |  |  |
| 40 years and below | |  |  |  | 1.00 (Ref) | — |  |
| 41 to 70 years | |  |  |  | 0.51 | 0.49, 0.53 | **<0.001** |
| 71+ years | |  |  |  | 0.30 | 0.28, 0.32 | **<0.001** |
| COVID-19 infection prior to December 15^th^ 2022 † | | | | 252,433 |  |  |  |
| No previous infection | | | |  | 1.00 (Ref) | — |  |
| Alpha/Beta/Gamma infection (before 1^st^ July 2021) | | | |  | 0.70 | 0.64, 0.77 | **<0.001** |
| Delta infection (after 1^st^ July 2021) | | | |  | 0.28 | 0.20, 0.37 | **<0.001** |
