# Supplementary material for: Hybrid immunity and protection against infection during the Omicron wave in Malta
Source: Emerg Microbes Infect. 2023 Jan 2;12(1):e2156814. doi: 10.1080/22221751.2022.2156814 (PMC9817114; doi:10.1080/22221751.2022.2156814)
Supplement: Supplemental Material [file TEMI_A_2156814_SM5900.zip › Supplementary Table 2.docx]

**Supplementary Table 2 : Counts and percentages of combinations of doses, infections and time stratified by age groups**

| **Characteristic** | | | | **40 years and below** | | **41 years - 70 years** | | **71+ years** | |
| --- | --- | --- | --- | --- | --- | --- | --- | --- | --- |
| Combination of doses, infection, and time | | | |  |  |  |  |  |  |
| ***Doses*** | ***Time from latest vaccination to 15th December 2021*** | ***Infection before 15^th^ December 2021*** | ***Time from latest infection to 15th December 2021*** | ***N = 93,769*** | | ***N = 103,549*** | | ***N = 55,116*** | |
| 2 | >20 weeks | No | *NA* | 41577 | 44.30% | 17739 | 17.10% | 2374 | 4.30% |
| 2 | >20 weeks | Yes | >20 weeks | 2662 | 2.80% | 871 | 0.80% | 145 | 0.30% |
| 2 | >20 weeks | Yes | <20 weeks | 518 | 0.60% | 260 | 0.30% | 24 | 0.00% |
| 2 | <20 weeks | No | *NA* | 28307 | 30.20% | 6345 | 6.10% | 629 | 1.10% |
| 2 | <20 weeks | Yes | >20 weeks | 1255 | 1.30% | 272 | 0.30% | 27 | 0.00% |
| 2 | <20 weeks | Yes | <20 weeks | 198 | 0.20% | 67 | 0.10% | 12 | 0.00% |
| 3 | >20 weeks | No | *NA* |  |  |  |  |  |  |
| 3 | >20 weeks | Yes | >20 weeks |  |  |  |  |  |  |
| 3 | >20 weeks | Yes | <20 weeks |  |  |  |  |  |  |
| 3 | <20 weeks | No | *NA* | 17319 | 18.50% | 73090 | 70.60% | 49388 | 89.60% |
| 3 | <20 weeks | Yes | >20 weeks | 1700 | 1.80% | 4338 | 4.20% | 2221 | 4.00% |
| 3 | <20 weeks | Yes | <20 weeks | 232 | 0.20% | 567 | 0.50% | 296 | 0.50% |
