# Supplementary material for: Hybrid immunity and protection against infection during the Omicron wave in Malta
Source: Emerg Microbes Infect. 2023 Jan 2;12(1):e2156814. doi: 10.1080/22221751.2022.2156814 (PMC9817114; doi:10.1080/22221751.2022.2156814)
Supplement: Supplemental Material [file TEMI_A_2156814_SM5900.zip › Supplementary Table 3.docx]

| Filtering Process | Number |
| --- | --- |
| **Rows (individual vaccination events until 8^th^ March 2022)** |  |
| original vaccination events listed | 1285371 |
| Removing all test and erronous IDs | 1269290 |
| Removing erronous vaccine date entries | 1237765 |
| Removing erronous children vaccination dates | 1237028 |
| **Individual records** |  |
| Number of vaccination records | 464053 |
| Removing those with 4 doses or more | 459128 |
| Removing those with incorrect 3rd dose date | 458035 |
| Removing those with 2 JnJ doses | 457201 |
| Removing incorrect Pfizer/Biontech date entries <21 days between doses | 443736 |
| removing incorrect Oxford-AstraZeneca dates <56 days between doses | 440554 |
| removing incorrect Moderna dates <21 days between doses | 440296 |
| removing incorrect JnJ entries (administration prior to actual starting date for JnJ) | 440156 |
| removing incorrect booster entries (<21 days from 2nd dose) | 439871 |
| removing JNJ as second dose | 439767 |
| Removing foreign vaccinations and incorrect combinations of vaccine | 438881 |
|  |  |
| Removing those with a 3rd dose after 15th Dec | **252433** |

**Supplementary Table 3: The filtering and cleaning process applied to original dataset**
