# Supplementary material for: Hybrid immunity and protection against infection during the Omicron wave in Malta
Source: Emerg Microbes Infect. 2023 Jan 2;12(1):e2156814. doi: 10.1080/22221751.2022.2156814 (PMC9817114; doi:10.1080/22221751.2022.2156814)

**Supplementary Table 4**

**Sensitivity Analysis comparing Forest Plots for different dates for when Omicron became the dominant variant in Malta.**

The following three forest plots compare odds ratios for 8^th^, 15^th^ and 22^nd^ December 2021. In all cases the trend is similar, and 15^th^ December,2021 was chosen as the preferred date as a midpoint for the three dates. It is also the middle of the week when Omicron became the dominant variant in Malta.

There is a missing entry for 22^nd^ December 2021 for 2 doses, <20 weeks since vaccination, Previously infected and <20 weeks since infection because there were no vaccinees in the Astrazeneca vaccine category for that category on the 22^nd^ of December 2021.

**8^th^ December 2021 (1 week before the selected date of 15^th^ December 2021)**


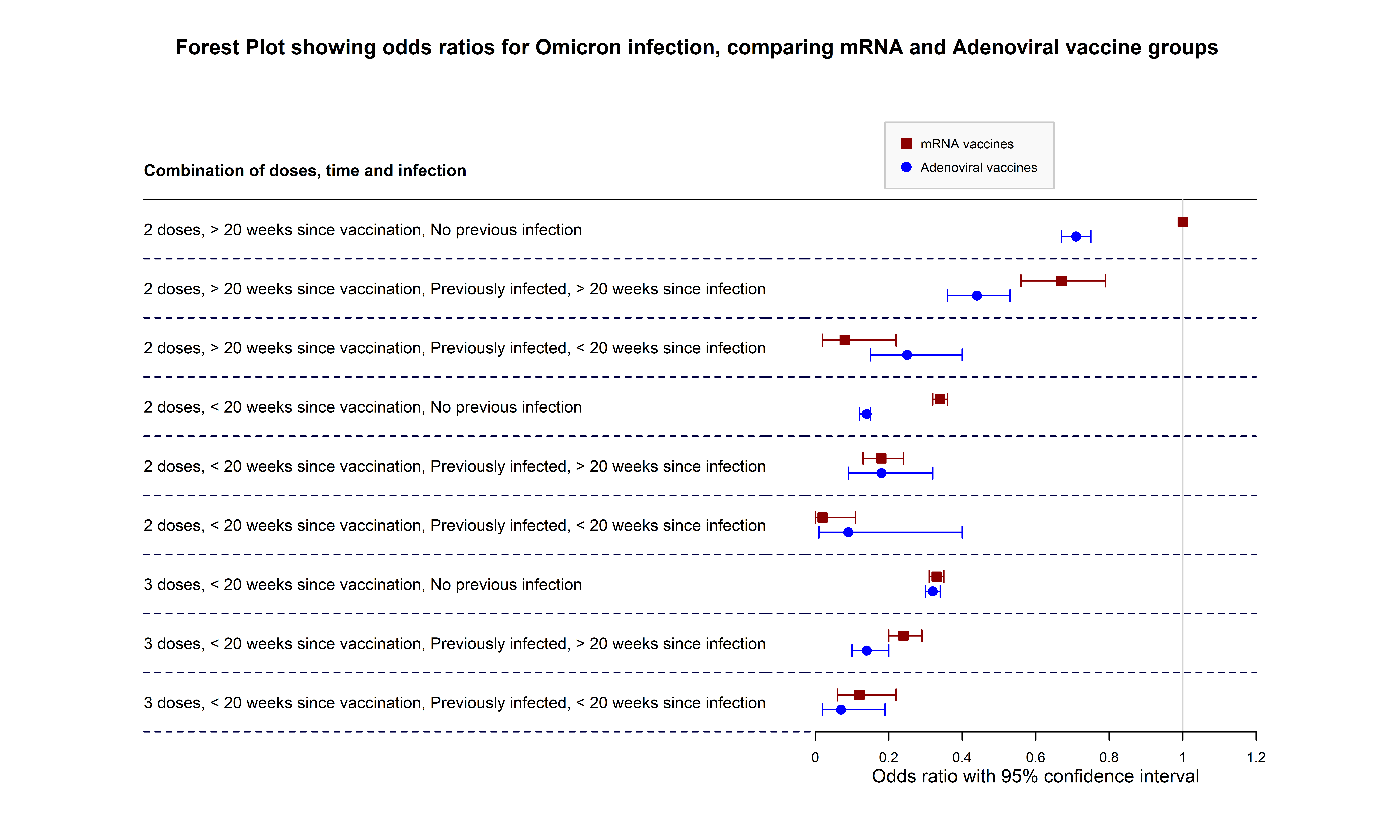


**15^th^ December 2021 (date selected for this study)**


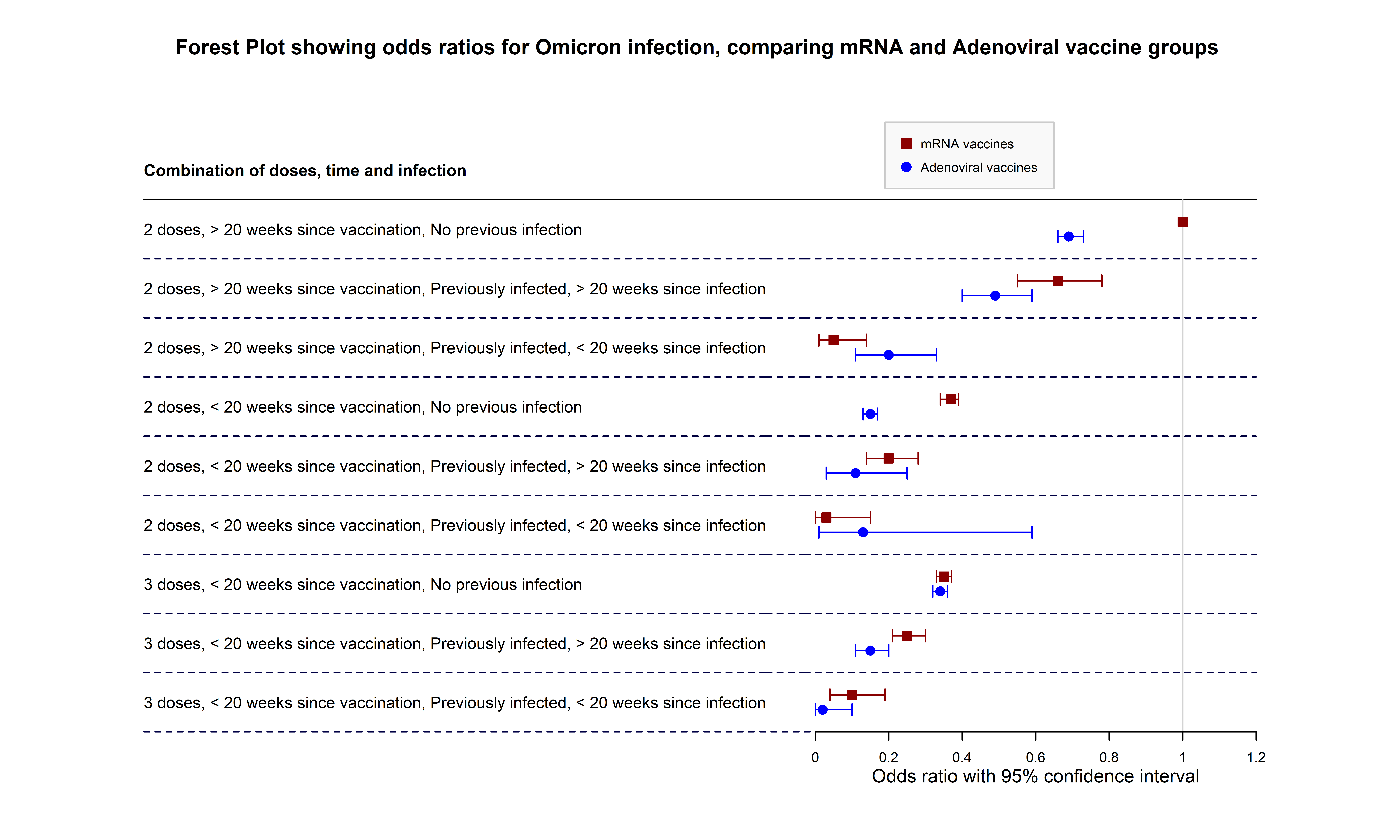


**22^nd^ December 2021**


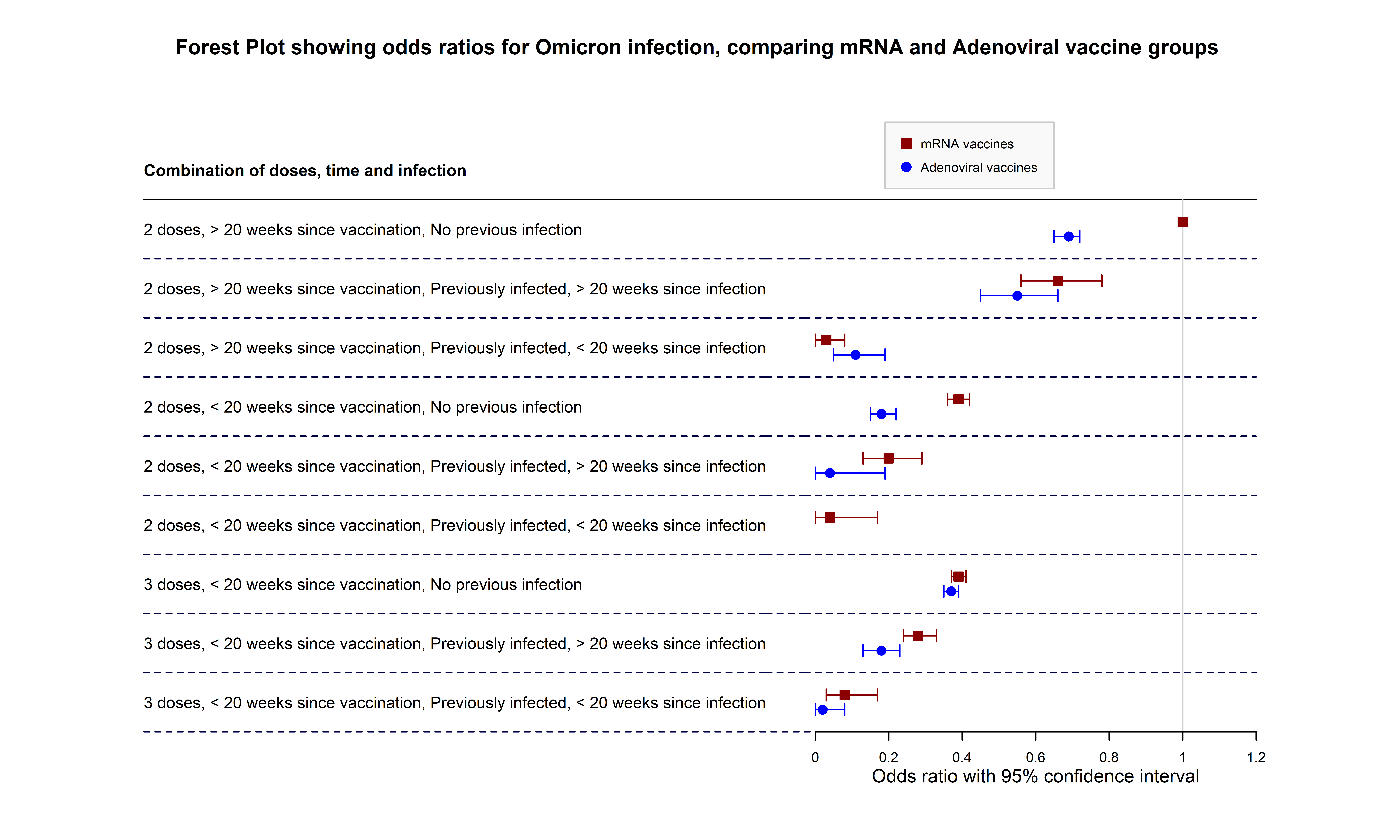

Supplement: Supplemental Material [file TEMI_A_2156814_SM5900.zip › Supplementary Table 4.docx]
